# Dysnatremia, its correction, and mortality in patients undergoing continuous renal replacement therapy: a prospective observational study

**DOI:** 10.1186/s12882-015-0215-1

**Published:** 2016-01-05

**Authors:** Seung Seok Han, Eunjin Bae, Dong Ki Kim, Yon Su Kim, Jin Suk Han, Kwon Wook Joo

**Affiliations:** Department of Internal Medicine, Seoul National University College of Medicine, 101 Daehakro, Jongno-gu, Seoul 03080 South Korea; Kidney Research Institute, Seoul National University College of Medicine, 03080 Seoul, South Korea

**Keywords:** Continuous renal replacement therapy, Dysnatremia, Hypernatremia, Hyponatremia, Mortality

## Abstract

**Background:**

Although dysnatremia has been reported to be correlated with mortality risk, this issue remains unresolved in patients undergoing continuous renal replacement therapy (CRRT). Furthermore, it has not been determined whether change in or correction of sodium is related to mortality risk in this subset.

**Methods:**

A total of 569 patients were prospectively enrolled at the start of CRRT between May 2010 and September 2013. The patients were divided into 5 groups: normonatremia (135–145 mmol/L), mild hyponatremia (131.1–134.9 mmol/L), moderate to severe hyponatremia (115.4–131.0 mmol/L), mild hypernatremia (145.1–148.4 mmol/L), and moderate to severe hypernatremia (148.5–166.0 mmol/L). The non-linear relationship between sodium and mortality was initially explored. Subsequently, the odds ratios (ORs) for 30-day mortality were calculated after adjustment of multiple covariates.

**Results:**

The relationship between baseline sodium and mortality was U-shaped. The mild hyponatremia, moderate to severe hyponatremia, and moderate to severe hypernatremia groups had greater ORs for mortality (1.65, 1.91, and 2.32, respectively) than the normonatremia group (all *P* values < 0.05). However, later sodium levels (24 and 72 h after CRRT) did not predict 30-day mortality. Furthermore, the changes in sodium over 24 or 72 h, including the appropriate correction of dysnatremia, did not show any relationships with mortality, irrespective of baseline sodium level.

**Conclusions:**

Sodium level at the start of CRRT was a strong predictor of mortality. However, changes in sodium level and the degree of sodium correction were not associated with the mortality risk in the patients with CRRT.

**Electronic supplementary material:**

The online version of this article (doi:10.1186/s12882-015-0215-1) contains supplementary material, which is available to authorized users.

## Background

Both hyponatremia and hypernatremia are important risk factors for high morbidity and mortality in several clinical settings [1, 2]. However, reports have conflicted about the dependent and independent relationships of sodium with mortality. A relationship is plausible because both hyponatremia and hypernatremia have adverse consequences via their effects on the brain, heart, and other organs [3, 4]. However, dysnatremia might be only a marker of comorbidities, with deaths attributable mainly to the severity of the diseases and not to dysnatremia itself [5]. A recent review of hyponatremia cases concluded that deaths were associated with the underlying illness, rather than with the severity of hyponatremia [6]. Given the nature of this issue, it is unclear whether the correction of sodium can reduce the mortality risk. Most randomized controlled trials of drugs for sodium correction have not demonstrated a survival advantage [7]. Although these results have not excluded the need for sodium correction, it is necessary to address whether sodium correction alone can reduce mortality. A well-designed observational study could be helpful because randomized controlled trials for this issue are not feasible.

Continuous renal replacement therapy (CRRT) is increasingly used in severe acute kidney injury (AKI), because it can easily control biochemical imbalances due to AKI [8]. The presence of AKI or comorbidities hinders the effectiveness of conventional fluid or drug management, but CRRT can overcome these difficulties and achieve normonatremia. Here, we first addressed how sodium levels predict mortality in a prospective cohort of patients receiving CRRT, in whom the control of dysnatremia was extremely difficult with conventional treatment alone. Whether sodium correction is independently associated with reduced mortality in comorbid conditions was also addressed.

## Methods

### Participants and data collection

Data on patients undergoing CRRT were obtained prospectively from a cohort from the tertiary referral center (Seoul National University Hospital). The inclusion criteria were as follows: CRRT was needed due to severe AKI; and the patient’s age was 18 years old or older. Accordingly, a total of 669 patients were enrolled at the start of CRRT from May 2010 to September 2013. All of the patients who participated in the present study provided informed consent and agreed with serial examination of serum sodium in adherence to the study protocol. We excluded patients who were previously diagnosed with end-stage renal disease or who received dialysis before enrollment (*n* = 97). If the patients underwent CRRT more than once (*n* = 3), only the first experience was counted as a single case. Consequently, 569 patients were analyzed for the present study. Flow diagram for this process is shown in Additional file 1.

Clinical parameters, such as age, sex, weight, cause of AKI, dialysis dose, the need for mechanical ventilation, the use of vasoactive drugs, and underlying chronic kidney disease, were recorded at the start of CRRT. The causes of AKI were divided into sepsis, surgery, nephrotoxin, and others. Information on comorbidities was collected and was presented as Charlson Comorbidity Index [9]. The Acute Physiology and Chronic Health Evaluation (APACHE) ІІ score was calculated to quantitatively assess each patient’s status [10]. Serum sodium, creatinine, and albumin were measured (Toshiba-200FR, Toshiba, Tokyo, Japan), and the urine output data during the first 2 h of CRRT were recorded. Serum sodium was additionally measured at 24 and 72 h after CRRT. Fluid balance before evaluation of later sodium levels (24 and 72 h) was monitored and expressed as follows: fluid balance = total input – total output. There were no missing data for any of the variables. Corrected sodium values for serum glucose were used in the study analyses, based on the following formula: corrected sodium = measured sodium + 0.016 × (serum glucose – 100) [11]. The onsets of dysnatremia were presented as acute (duration < 48 h), chronic (≥ 48 h), or unknown duration. If dysnatremia was identified, it was treated using CRRT with diluted or concentrated substitution fluid to control the speed of correction [12, 13].

The primary outcome was 30-day all-cause mortality after starting CRRT. The mortality data were obtained from the national database of Statistics Korea; all Koreans have a resident registration number and death events are recorded and stored at a national level.

The study protocol complies with the Declaration of Helsinki and received full approval from the institutional review board at the Seoul National University Hospital (no. 1309-018-518). No extramural funding was used to support this work. The authors are solely responsible for the design and conduct of this study, all study analyses and drafting and editing of the paper.

### Statistical analysis

All of the analyses and calculations were performed using STATA software (version 12.0, StataCorp LP, College Station, Texas, USA). The data are presented as the mean ± standard deviations for continuous variables and as proportions for categorical variables. Based on variable distributions using histograms, the variables with non-normal distributions are expressed as the median (interquartile ranges). The chi-squared test was used to compare the categorical variables. Comparisons between normally distributed continuous variables were performed using an analysis of variance or a post-hoc analysis of least significant difference, according to the number of comparison groups. Comparisons between non-normally distributed continuous variables were performed by either the Kruskal-Wallis test or the Mann–Whitney *U* test, depending on the number of comparison groups. The dose–response relationship (in particular, the non-linear relationship) between sodium and mortality was initially explored by a restricted cubic spline analysis. Due to the U-shaped relationship, we divided the patients into five groups as follows: normonatremia (135–145 mmol/L), mild hyponatremia, moderate to severe hyponatremia, mild hypernatremia, and moderate to severe hypernatremia. The mild and moderate to severe dysnatremia groups were determined based on the median sodium value within each hypo- and hyper-natremia group. Survival curves were drawn using the Kaplan-Meier method. To compare survival curves between groups, the log-rank test was initially applied. The odds ratios (ORs) and 95 % confidence intervals (CIs) for 30-day mortality were calculated using the logistic regression model after adjustment of all covariates (as provided in Table 1). A *P* value less than 0.05 was considered significant.

**Table 1:** Baseline characteristics and laboratory findings of the patients at the time of admission to the intensive care unit

	Sodium group					
	Moderate to severe hyponatremia (*n* = 111)	Mild hyponatremia (*n* = 112)	Normonatremia (*n* = 247)	Mild hypernatremia (*n* = 49)	Moderate to severe hypernatremia (*n* = 50)	Total (*n* = 569)
Age (years)	63.2 ± 14.94	64.3 ± 15.37	63.9 ± 14.38	60.9 ± 16.15	59.7 ± 17.46	63.2 ± 15.14
Male sex (%)	56.8	61.6	62.3	61.2	56.0	60.5
Enrollment year (%)						
2010	15.3	13.4	14.6	14.3	10.0	14.1
2011	29.7	33.0	32.4	16.3	32.0	30.6
2012	33.3	34.8	35.6	46.9	40.0	36.4
2013	21.6	18.8	17.4	22.4	18.0	19.0
Body weight (kg)	62.2 ± 11.56	64.3 ± 13.19	63.6 ± 14.02	65.5 ± 12.48	62.3 ± 10.62	63.5 ± 13.00
Cause of acute kidney injury (%)*						
Sepsis	44.1 †	55.4	49.4	55.1	36.0*	48.9
Surgery	3.6	6.2	11.7	8.2	8.0	8.4
Nephrotoxin	5.4	4.5	8.1	6.1	4.0	6.3
Other	46.8	33.9	30.8	30.6	52.0	36.4
Dialysis dose (mL/kg/hour)	41.9 ± 15.43	47.7 ± 18.61	45.0 ± 16.99	46.2 ± 19.19	41.7 ± 15.90	44.8 ± 17.21
Need for MV (%)	76.6	83.9	85.0	93.9	80.0	83.5
Use of vasoactive drugs (%)	73.0	68.8	72.1	67.3	74.0	71.4
Chronic kidney disease (%)	48.6	46.4	42.5	26.5*	34.0	42.4
Charlson Comorbidity Index †	3.5 ± 2.18 †	3.3 ± 2.25*	2.7 ± 2.13	2.5 ± 2.05	2.5 ± 1.98	2.9 ± 2.17
APACHE II score*	29.6 ± 9.56	28.3 ± 7.91	28.7 ± 8.00	30.6 ± 8.05	32.5 ± 8.15 †	29.3 ± 8.38
Blood findings						
Sodium (mmol/L) ‡	126.6 ± 3.99 ‡	133.2 ± 1.09 ‡	139.5 ± 2.74	146.8 ± 0.92 ‡	154.0 ± 4.81 ‡	137.6 ± 8.27
Creatinine (mg/dL)*	3.3 ± 2.01	3.7 ± 2.67 †	3.1 ± 1.61	2.8 ± 1.27	2.9 ± 1.69	3.2 ± 1.94
Albumin (g/dL)	2.7 ± 0.57	2.6 ± 0.52	2.7 ± 0.52	2.6 ± 0.51	2.7 ± 0.50	2.7 ± 0.53
Glucose (mg/dL) †	139 (108–190)	147 (116–196)	153 (113–229)	164 (119–222)	191 (134–311) †	152 (115–218)
Urine output (mL/2 hours)	15 (0–70)	20 (0–55)	20 (0–90)	30 (0–118)	28 (4–64)	20 (0–78)
Fluid balance						
During 24 h (mL) ‡	808.3 ± 1527.1861	1069.0 ± 2242.01	1250.7 ± 1971.14	1736.9 ± 2378.86	2772.3 ± 2555.26 ‡	1304.2 ± 2103.42
During 72 h (mL) †	816.1 ± 2966.11	1036.9 ± 3773.28	999.2 ± 2854.91	1957.4 ± 3839.81	2611.9 ± 2918.75 ‡	1185.9 ± 3197.57
Onset of dysnatremia (%)						
Acute	19.8	34.8	–	77.6	48.0	38.2^§^
Chronic	61.3	46.4	–	10.2	30.0	43.5
Unknown	18.9	18.8	–	12.2	22.0	18.3
Follow-up duration (days)	13 (4–49)*	19 (4–123)	29 (5–273)	23 (5–434)	9 (3–93)*	19 (4–193)

Comparisons were evaluated using the chi-squared test for categorical variables, the ANOVA test for normally distributed continuous variables (post hoc analysis of LSD between two groups), and the Kruskal-Wallis test for non-normally distributed continuous variables (Mann–Whitney *U* test between two groups). The normonatremia group served as a reference for comparison between two groups

* *P* < 0.05; † *P* < 0.01; ‡ *P* < 0.001; § proportion among dysnatremia group

*MV* mechanical ventilation, *APACHE* acute physiology and chronic health evaluation

## Results

### Baseline characteristics

The baseline characteristics of the patients are shown and compared between groups with respect to serum sodium level in Table 1. For the 569 subjects, the mean age was 63 years. Except for one Caucasian subject, all other patients were Asian. Approximately half received CRRT because of sepsis. The ranges of sodium levels in dysnatremia groups were as follows: mild hyponatremia, 131.1–134.9 mmol/L; moderate to severe hyponatremia, 115.4–131.0 mmol/L; mild hypernatremia, 145.1–148.4 mmol/L; and moderate to severe hypernatremia, 148.5–166.0 mmol/L. The subjects were followed for a median of 19 days (4 to 193 days).

The AKI causes differed among sodium groups. Hyponatremia groups had higher scores for the Charlson Comorbidity Index than the normonatremia group. The moderate to severe hypernatremia group had higher APACHE II scores than the normonatremia group. The serum creatinine and albumin levels were similar among the sodium groups, except for a higher creatinine level in the mild hyponatremia group. Treatment with hypertonic saline was used in 8 patients, of whom 7 had moderate to severe hyponatremia and 1 had mild hyponatremia. Hypotonic saline was used in 13 patients with mild hypernatremia and 16 patients with moderate to severe hypernatremia.

### Risk of mortality according to sodium level

**Fig. 1:**
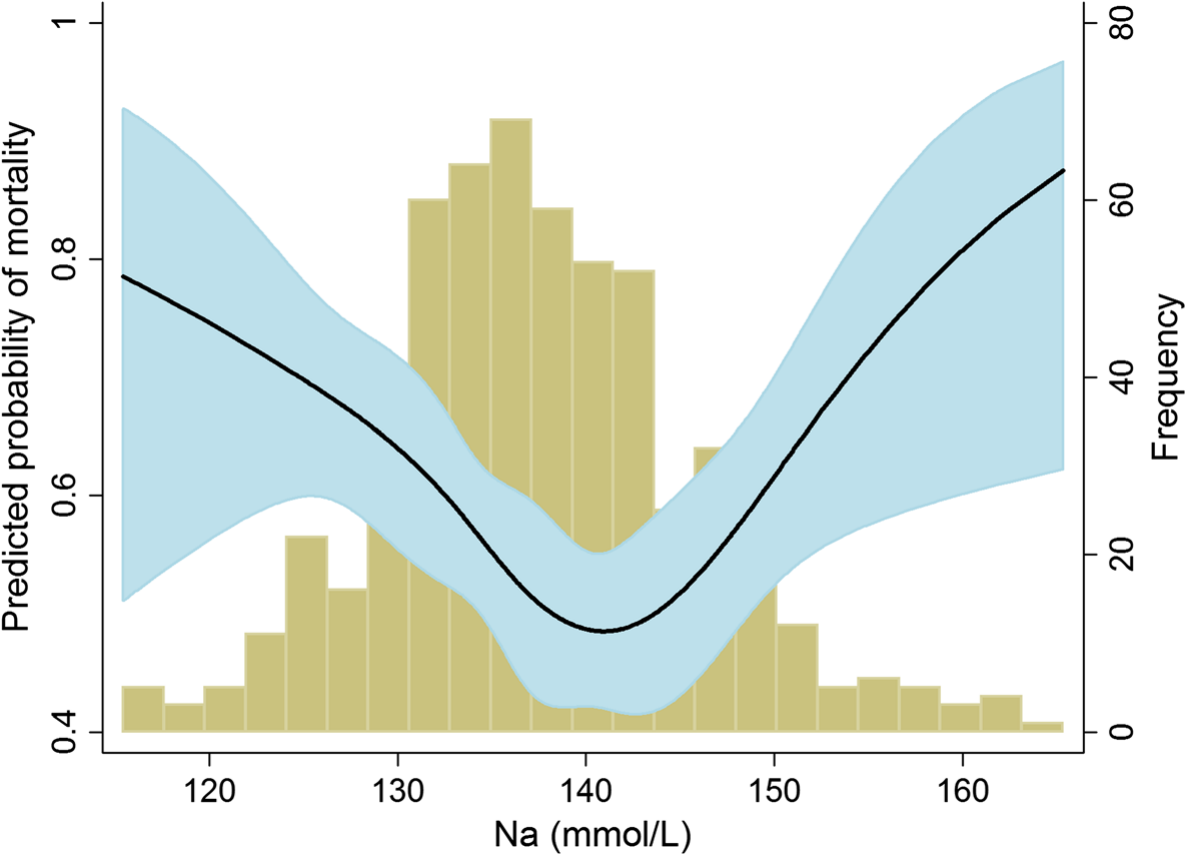
Non-linear relationship between baseline sodium level and the predicted probability of 30-day mortality. The range area indicates 95 % confidence intervals. A histogram of the sodium level is also shown

**Fig. 2:**
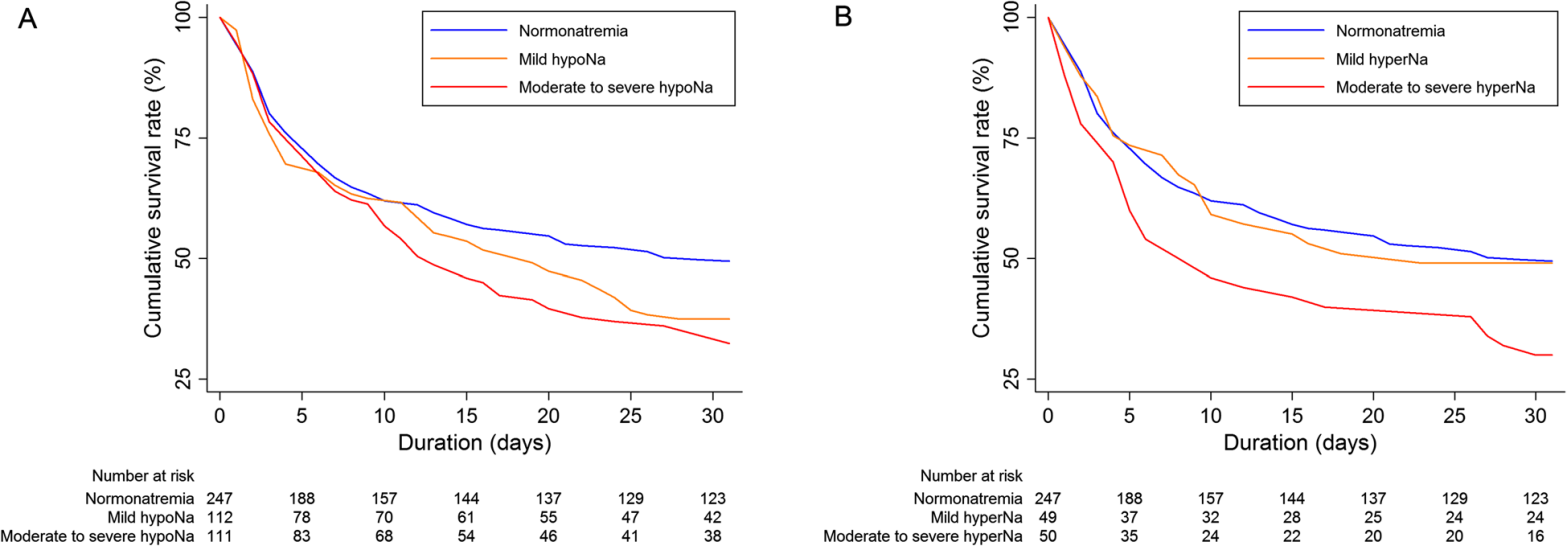
Kaplan-Meier survival curves according to the presence of hyponatremia (**a**) or hypernatremia (**b**)

**Fig. 3:**
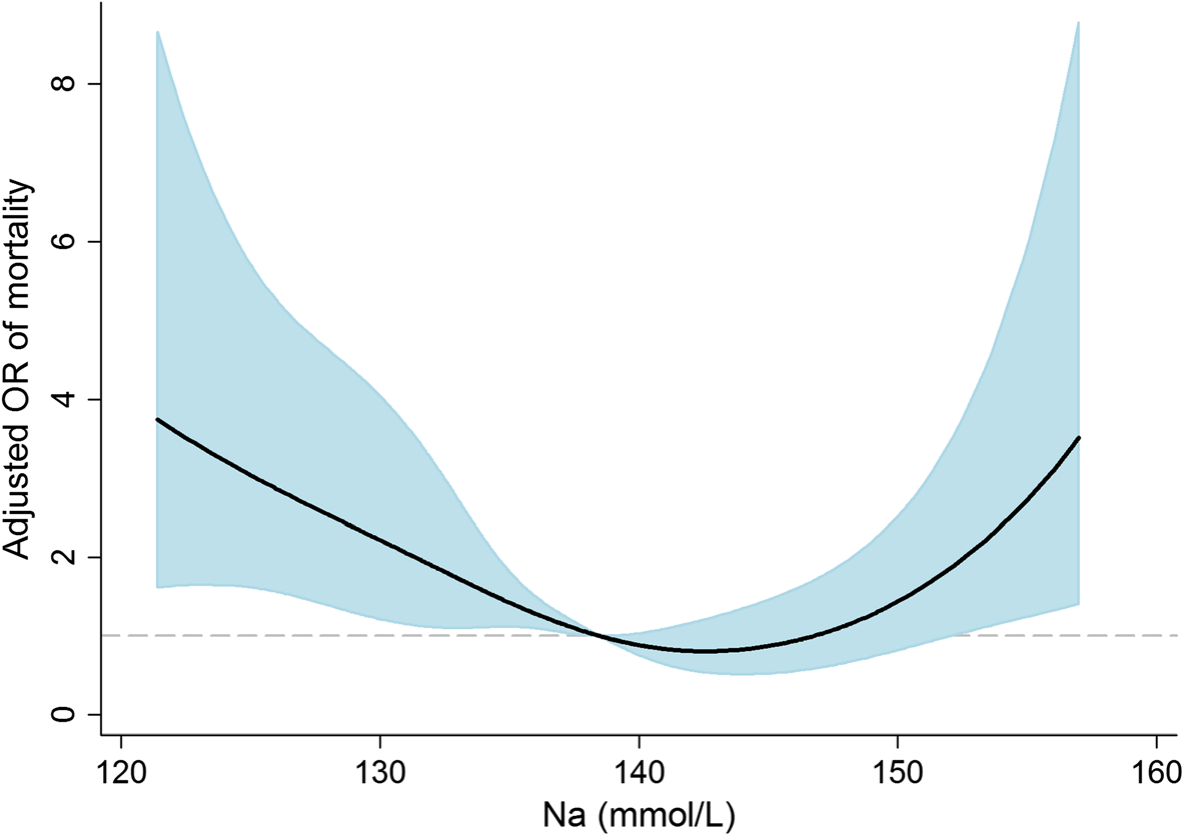
Non-linear relationship between baseline sodium level and the odds ratio of 30-day mortality after adjustment for multiple covariates. The range area indicates 95 % confidence intervals

A total of 57.5 % of the subjects died within 30 days after starting CRRT. Figure 1 shows the relationship between baseline sodium level and the predicted probability of mortality. The relationship between these variables appeared to be U-shaped. Based on this observation, we divided the patients into 5 groups, from hyponatremia to hypernatremia, and we used the normonatremia group as the reference for all of the analyses. The crude 30-day mortality rate was as follows: 50.2 % of those with normonatremia, 62.5 % with mild hyponatremia, 65.8 % with moderate to severe hyponatremia, 51.0 % with mild hypernatremia, and 70.0 % with moderate to severe hypernatremia. Kaplan-Meier survival curves (Fig. 2) show that the survival rates were significantly different (*P* = 0.016 among the 5 groups, *P* = 0.024 among the Fig. 1a groups, and *P* = 0.051 among the Fig. 1b groups). The risks of 30-day mortality according to sodium group are shown in Table 2. In univariate analysis, both hypo- and hypernatremia groups, with the exception of the mild hypernatremia group, had greater ORs for mortality than the normonatremia group. This trend remained significant irrespective of covariates' effects. Similar to these results, the correlation between sodium levels and ORs of mortality after adjustment for all of the covariates was U-shaped (Fig. 3). After initiating CRRT, 542 and 457 patients survived at 24 and 72 h, respectively, and their sodium levels were included in the subsequent analyses. However, sodium levels at 24 and 72 h were not associated with mortality risk (Table 3). Additionally, when adjusted for the sodium level at baseline, the later sodium levels did not show a significant correlation with mortality (all *P*s > 0.05).

**Table 2:** Odds ratios for 30-day mortality according to the concentration of serum sodium

	Univariate		Multivariate^a^	
Group	OR (95 % CI)	*P*	OR (95 % CI)	*P*
Moderate to severe hypoatremia	1.91 (1.197–3.033)	0.007	3.61 (1.438–9.049)	0.006
Mild hyponatremia	1.65 (1.047–2.610)	0.031	2.45 (1.051–5.705)	0.038
Normonatremia	1 (Reference)		1 (Reference)	
Mild hypernatremia	1.03 (0.560–1.908)	0.917	1.06 (0.461–2.438)	0.890
Moderate to severe hypernatremia	2.32 (1.203–4.453)	0.012	2.94 (1.119–7.722)	0.029

^a^Adjusted for age, sex, enrollment year, weight, cause of acute kidney injury, dialysis dose, need for mechanical ventilation, use of vasoactive drugs, chronic kidney disease, Charlson Comorbidity Index, APACHE II score, creatinine, albumin, urine output, fluid balance, and onset of dysnatremia

*OR* odds ratio, *CI* confidence interval

**Table 3:** Adjusted odds ratios for 30-day mortality according to sodium level after 24 and 72 h

	24-h sodium		72-h sodium	
Group	OR (95 % CI)^a^	*P*	OR (95 % CI)^a^	*P*
Moderate to severe hyponatremia	0.81 (0.458–1.418)	0.454	0.89 (0.524–1.528)	0.683
Mild hyponatremia	1.08 (0.629–1.868)	0771	1.08 (0.637–1.825)	0.780
Normonatremia	1 (Reference)		1 (Reference)	
Mild hypernatremia	0.90 (0.270–3.027)	0.869	4.69 (0.835–26.302)	0.079
Moderate to severe hypernatremia	6.33 (0.794–50.382)	0.081	3.13 (0.606–16.156)	0.173

^a^Adjusted for age, sex, enrollment year, weight, cause of acute kidney injury, dialysis dose, need for mechanical ventilation, use of vasoactive drugs, chronic kidney disease, Charlson Comorbidity Index, APACHE II score, creatinine, albumin, urine output, fluid balance, and onset of dysnatremia

*OR* odds ratio, *CI* confidence interval

### Sodium correction and mortality

**Fig. 4:**
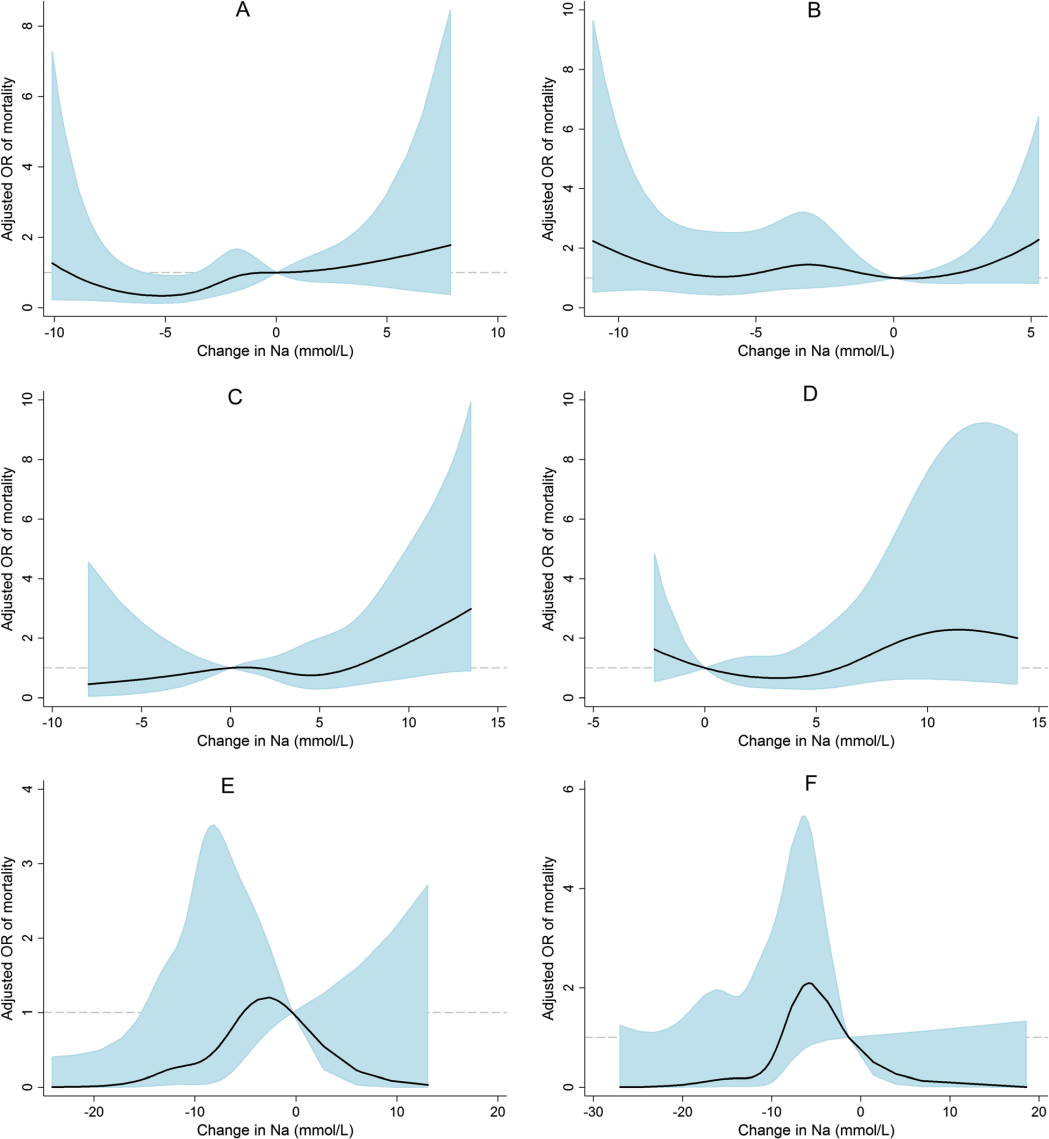
Non-linear relationships between the adjusted odds ratio of 30-day mortality and changes in sodium in the normonatremia (**a**, **b**), hyponatremia (**c**, **d**), and hypernatremia (**e**, **f**) groups. Figures (**a**), (**c**), and (**e**) show the relationships with changes over 24 h. Figures (**b**), (**d**), and (**f**) show the relationships with changes over 72 h

We further analyzed the risk of mortality according to the change in sodium over 24 or 72 h. The number of 30-day death cases for each group is shown in Additional file 2. First, the non-linear relationships between adjusted ORs for mortality and changes in sodium levels were explored (Fig. 4). In the groups with baseline normonatremia or hyponatremia, the change in sodium over 24 or 72 h did not show an association with the risk of mortality (Fig. 4a to d). In the group with baseline hypernatremia (Fig. 4e and f), a greater decrease in sodium seemed to incur a reduced risk of mortality. However, this trend was shown only in the change over 24 h and not over 72 h. In particular, comparison of mortality rates within three sodium groups, i.e., normonatremia to normonatremia, hyponatremia to hyponatremia, and hypernatremia to hypernatremia, showed no significant difference (Additional file 3). Moreover, when adjusted for the sodium level at baseline, the change in sodium levels did not have any significance with mortality (all *P*s > 0.05). These results indicated that sodium change and correction of dysnatremia did not change the mortality risk.

## Discussion

The present study enrolled patients with specific characteristics (extremely severe AKI and high mortality and morbidity risks); thus, CRRT was the preferred method for correction of imbalances in biochemical parameters, including dysnatremia. The results report for the first time a correlation between baseline sodium level and mortality risk in a group undergoing CRRT. However, the change in sodium, as a marker of sodium correction, was not associated with mortality risk. These analyses were helpful in determining whether the correction of dysnatremia independently reduces mortality in the presence of several comorbidities.

Dysnatremia is associated with various negative consequences, such as decreased brain function [3], compromised cardiac contractility [4], increased insulin resistance [14], abnormalities in neuromuscular function [15], creation of an inflammatory milieu [16], and aggravation of interstitial edema [17]. Based on these pathologic conditions, it is plausible that dysnatremia contributes to mortality risk. The present study demonstrated that dysnatremia predicted mortality in a CRRT cohort. For patients undergoing CRRT, any of the negative conditions driven by dysnatremia could have contributed to the high mortality.

Similar to the present results with CRRT, dysnatremia has been associated with high mortality in other several clinical settings, even with normal kidney function [18]. Most studies adjusted for various comorbidities in their analyses, thus suggesting an independent association between sodium and mortality. However, this methodological approach is not sufficient to draw a final conclusion. Other important data are needed, including whether each death is directly related to dysnatremia, and whether the reduction or treatment of dysnatremia itself reduces mortality. A previous analysis of cases with severe hyponatremia indicated that deaths were primarily caused by underlying illness and not by hyponatremia itself [6]. Furthermore, two thirds of those patients died, although their sodium levels returned to normal or near normal ranges. No studies have investigated the effects of sodium correction on mortality by using randomization of participants and controlling for confounders.

In addition to the correlation issue between dysnatremia and mortality, it remains unresolved whether the correction of sodium could reduce mortality, particularly in the presence of several comorbidities. A study of this issue is difficult to conduct. Thus, the present study might be helpful, despite its observational design, in understanding the correlation. In contrast to the baseline level, subsequent sodium levels did not predict mortality irrespective of timeframe (24 and 72 h), possibly because of case-mix results, such as the group with baseline dysnatremia later having normonatremia. Furthermore, the change in sodium level, including the appropriate correction of dysnatremia, was not correlated with mortality. These results suggested that sodium level itself might accurately predict mortality in association with comorbidities or other related factors; however, the correction of sodium alone did not contribute to a survival benefit, particularly under the strong influence of comorbidities.

The association with mortality may differ for hyponatremia and hypernatremia. In the present cohort, mild hypernatremia was not associated with mortality in contrast to the results of mild hyponatremia. The discrepancy between the effects of hypo- and hypernatremia has been reported in other observational studies [19–21]. Each association with mortality could be sensitive to the disease state of study. However, although the baseline characteristics differ across studies, hyponatremia and hypernatremia should be considered separately in research, as well as in clinical practice.

Although the present results are informative, this study has some limitations. First, the observational study design limits the drawing of conclusions, because of causality. However, randomized trials of dysnatremia treatment to determine survival benefit are not feasible, particularly in the presence of comorbidities; thus, the present results might be helpful in raising awareness of the correlation between sodium correction and outcomes. Second, the present cohort of patients who underwent CRRT is a particular subgroup, thus hindering the applicability of the conclusions to other populations. Furthermore, a single therapeutic intervention (i.e., sodium correction) might not show a survival benefit under conditions of high comorbidity rates of CRRT patients [22]. Third, sodium levels corrected for glucose may not be accurate, because the correction factor may be altered by patient characteristics or high glucose states [23, 24]. Lastly, we did not retrieve important variables (e.g., correction rate of dysnatremia and neurologic outcomes), which could affect the overall outcome.

## Conclusions

Sodium level at the start of CRRT was a strong predictor of mortality, as previously observed in other clinical settings. However, the impact of sodium correction on mortality was not significant, and would have been weaker in the presence of multiple comorbidities or severe disease, both of which have been correlated with dysnatremia. Randomized controlled studies are needed to draw a firm conclusion, but the present study may be helpful in understanding the meaning of sodium levels and applying it in clinical practice.

## Additional files

Additional file 1:
**CONSORT flow diagram.** (PDF 171 kb) Additional file 2:
**Subject number according to the change of serum sodium during 24 or 72 hours.** (DOC 38 kb) Additional file 3:
**Adjusted odd ratios of mortality among the sodium groups such as normonatremia-to-normonatremia, hyponatremia-to-hyponatremia, and hypernatremia-to-hypernatremia.** (DOC 28 kb)

## Abbreviations

AKI: acute kidney injury
APACHE: acute physiology and chronic health evaluation
CI: confidence interval
CRRT: continuous renal replacement therapy
OR: odds ratio
