# Supplementary figures and images for: Dysnatremia, its correction, and mortality in patients undergoing continuous renal replacement therapy: a prospective observational study
Source: BMC Nephrol. 2016 Jan 5;17:2. doi: 10.1186/s12882-015-0215-1 (PMC4702339; doi:10.1186/s12882-015-0215-1)

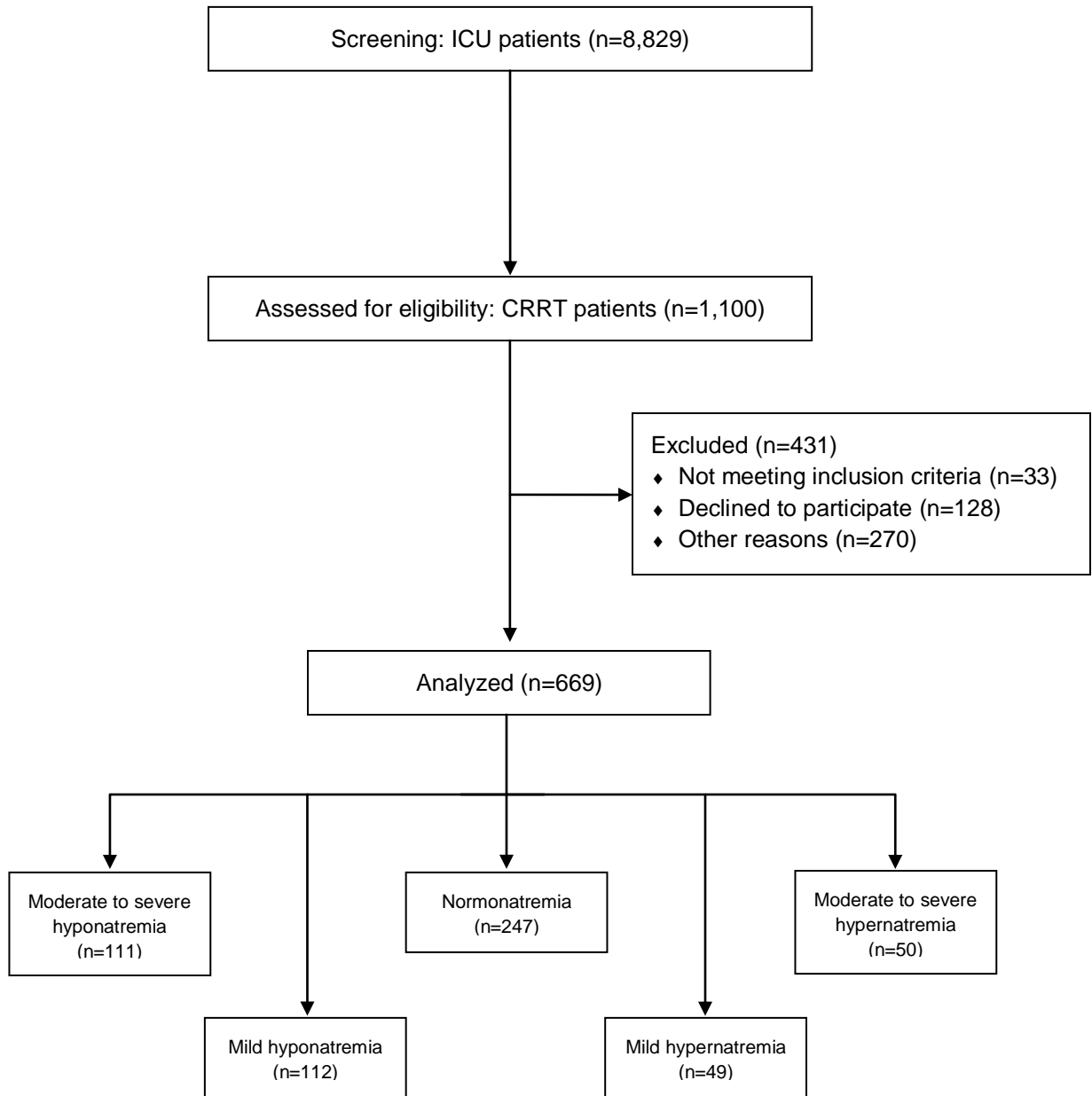

Supplement: Additional file 1: — CONSORT flow diagram. (PDF 171 kb) [file 12882_2015_215_MOESM1_ESM.pdf]
