# Supplementary material for: Dysnatremia, its correction, and mortality in patients undergoing continuous renal replacement therapy: a prospective observational study
Source: BMC Nephrol. 2016 Jan 5;17:2. doi: 10.1186/s12882-015-0215-1 (PMC4702339; doi:10.1186/s12882-015-0215-1)
Supplement: Additional file 2: — Subject number according to the change of serum sodium during 24 or 72 hours. (DOC 38 kb) [file 12882_2015_215_MOESM2_ESM.doc]

Additional file 2. Subject number according to the change of serum sodium during 24 or 72 hours

|  | At 24 hours (n = 542) | | | At 72 hours (n = 457) | | |
| --- | --- | --- | --- | --- | --- | --- |
| Baseline | Group | Total no. | No. of 30-day death cases (%) | Group | Total no. | No. of 30-day death cases (%) |
| Hyponatremia | Hyponatremia | 105 | 68 (64.8) | Hyponatremia | 77 | 39 (50.6) |
|  | Normonatremia | 106 | 63 (59.4) | Normonatremia | 93 | 52 (55.9) |
|  | Hypernatremia | 4 | 4 (100.0) | Hypernatremia | 4 | 3 (75.0) |
| Normonatremia | Hyponatremia | 47 | 17 (36.2) | Hyponatremia | 69 | 29 (42.0) |
|  | Normonatremia | 181 | 89 (49.2) | Normonatremia | 128 | 46 (35.9) |
|  | Hypernatremia | 7 | 6 (85.7) | Hypernatremia | 7 | 6 (85.7) |
| Hypernatremia | Hyponatremia | 4 | 1 (25.0) | Hyponatremia | 20 | 7 (35.0) |
|  | Normonatremia | 70 | 40 (57.1) | Normonatremia | 51 | 27 (52.9) |
|  | Hypernatremia | 18 | 12 (66.7) | Hypernatremia | 8 | 6 (75.0) |
