# Supplementary material for: Dysnatremia, its correction, and mortality in patients undergoing continuous renal replacement therapy: a prospective observational study
Source: BMC Nephrol. 2016 Jan 5;17:2. doi: 10.1186/s12882-015-0215-1 (PMC4702339; doi:10.1186/s12882-015-0215-1)
Supplement: Additional file 3: — Adjusted odd ratios of mortality among the sodium groups such as normonatremia-to-normonatremia, hyponatremia-to-hyponatremia, and hypernatremia-to-hypernatremia. (DOC 28 kb) [file 12882_2015_215_MOESM3_ESM.doc]

Additional file 3. Adjusted odd ratios of mortality among the sodium groups such as normonatremia-to-normonatremia, hyponatremia-to-hyponatremia, and hypernatremia-to-hypernatremia.

|  | 24 hours | | | 72 hours | | |
| --- | --- | --- | --- | --- | --- | --- |
| Group | n | OR (95% CI)* | *P* | n | OR (95% CI)* | *P* |
| Normo to normo | 181 | 1 (Reference) |  | 128 | 1 (Reference) |  |
| Hypo to hypo | 105 | 2.09 (0.841–5.172) | 0.113 | 77 | 2.05 (0.780–5.369) | 0.146 |
| Hyper to hyper | 18 | 1.96 (0.557–6.886) | 0.295 | 8 | 5.65 (0.895–35.686) | 0.066 |

*Adjusted for age, sex, enrollment year, weight, cause of acute kidney injury, dialysis dose, need for mechanical ventilation, use of vasoactive drugs, chronic kidney disease, Charlson Comorbidity Index, APACHE II score, creatinine, albumin, urine output, fluid balance, and onset of dysnatremia.

OR, odds ratio; CI, confidence interval.
